# Mediterranean Diet Modulates Gene Expression of Cholesterol Efflux Receptors in High‐Risk Cardiovascular Patients

**DOI:** 10.1002/mnfr.70050

**Published:** 2025-05-09

**Authors:** Javier Hernando‐Redondo, Álvaro Hernáez, Albert Sanllorente, Xavier Pintó, Ramón Estruch, Jordi Salas‐Salvadó, Dolores Corella, Fernando Arós, Miguel Ángel Martínez‐González, Isaac Subirana, Daniel Muñoz‐Aguayo, Mireia Malcampo, Lluis Serra‐Majem, Dora Romaguera, Jose Lapetra, Emilio Ros, Francisco Tinahones, Rosa Maria Lamuela‐Raventós, Enrique Gómez‐Gracia, Montserrat Fitó, Olga Castañer

**Affiliations:** ^1^ Consorcio CIBER, Pathophysiology of Obesity and Nutrition (CIBERobn) Instituto de Salud Carlos III Madrid Spain; ^2^ Unit of Cardiovascular Risk and Nutrition Hospital del Mar Medical Research Institute Barcelona Spain; ^3^ Ph.D. Program in Food Science and Nutrition University of Barcelona Barcelona Spain; ^4^ Blanquerna School of Health Sciences University Ramon Llull Barcelona Spain; ^5^ REGICOR Study Group Hospital del Mar Research Institute (IMIM) Barcelona Spain; ^6^ CIBER of Cardiovascular Diseases (CIBERCV) Instituto de Salud Carlos III (ISCIII) Madrid Spain; ^7^ Metropolitana Sud Research Support Unit Fundació Institut Universitari per a la recerca a l'Atenció Primària de Salut Jordi Gol i Gurina (IDIAPJGol) Hospitalet de Llobregat Barcelona Spain; ^8^ Metropolitana Sud Primary Care Institut Català de la Salut Hospitalet de Llobregat Barcelona Spain; ^9^ Lipids and Vascular Risk Unit, Internal Medicine Institut d'Investigació Biomèdica de Bellvitge (IDIBELL) Hospital Universitario de Bellvitge Universidad de Barcelona L'Hospitalet de Llobregat Spain; ^10^ Departament of Internal Medicine Institut d'Investigacions Biomèdiques August Pi i Sunyer (IDIBAPS) Hospital Clínic University of Barcelona Barcelona Spain; ^11^ Unit of Human Nutrition Department of Biochemistry and Biotechnology Institut d'Investigació Sanitària Pere Virgili (IISPV) Rovira i Virgili University Reus Spain; ^12^ Sant Joan de Reus University Hospital Institut d'Investigació Sanitària Pere Virgili Reus Spain; ^13^ Departament of Preventive Medicine University of Valencia Valencia Spain; ^14^ Cardiology department Organización Sanitaria Integrada Araba (OSI ARABA) University Hospital of Araba Victoria Spain; ^15^ University of País Vasco/Euskal Herria Unibersitatea (UPV/EHU) Vitoria‐Gasteiz Spain; ^16^ Department of Preventive Medicine and Public Health Universidad de Navarra Instituto de Investigación Sanitaria de Navarra (IdiSNA) Pamplona Spain; ^17^ REGICOR Study Group Hospital del Mar Medical Research Institute (IMIM) Barcelona Spain; ^18^ Institute for Biomedical Research University of Las Palmas de Gran Canaria Las Palmas de Gran Canaria Spain; ^19^ Research Group in Nutritional Epidemiology and Cardiovascular Pathophysiology Instituto de Investigación Sanitaria Illes Balears (IdISBa) Palma de Mallorca Palma Spain; ^20^ Departament of Family Medicine, Research Unity Distrito Sanitario Atención Primaria Sevilla Seville Spain; ^21^ Institut d'Investigacions Biomèdiques August Pi Sunyer (IDIBAPS) Hospital Clínic Barcelona Spain; ^22^ Departament of Endocrinology Hospital Virgen de la Victoria (IBIMA) University of Málaga Málaga Spain; ^23^ Department of Nutrition Food Science and Gastronomy School of Pharmacy and Food Sciences XaRTA, INSA University of Barcelona Barcelona Spain; ^24^ Department of Preventive Medicine and Public Health Instituto de Investigación Biomédica de Málaga‐IBIMA School of Medicine University of Málaga Malaga Spain; ^25^ CIBER de Epidemiología y Salud Pública (CIBERESP) Instituto de Salud Carlos III (ISCIII) Madrid Spain

**Keywords:** cardiovascular risk, Mediterranean diet, nutrigenomic, omics, randomized controlled trial, transcriptomic

## Abstract

In this study, we investigated gene expression related to cholesterol efflux receptors in individuals at high cardiovascular risk undergoing Mediterranean dietary interventions. Through transcriptomic analysis, we examined samples from two randomized controlled trials: PREDIMED and PREDIMED‐Plus, with 151 and 89 elderly adults, respectively. Blood cells were isolated at baseline and after a 12‐month intervention. In the PREDIMED trial, participants followed different Mediterranean diets: one supplemented with extra‐virgin olive oil (traditional Mediterranean diet enriched with extra‐virgin olive oil [MedDiet‐EVOO]), another with nuts (MedDiet enriched with nuts MedDiet‐Nuts [MedDiet‐Nuts]), and a low‐fat control diet. The PREDIMED‐Plus trial compared an energy‐reduced Mediterranean diet (Er‐MedDiet) with physical activity to an ad libitum Mediterranean diet. Over time, mild but significant upregulation of genes like ATP binding cassette subfamily A member 1 (*ABCA1*), retinoid X receptor alpha (*RXRA*), retinoid X receptor beta (*RXRB*), and *Nuclear Receptor Subfamily 1 Group H Member 3 (NR1H3)* was observed in response to MedDiet‐EVOO, MedDiet‐Nuts, and Er‐MedDiet. Notably, *RXRA* expression was higher in both MedDiet‐EVOO and MedDiet‐Nuts compared to the control diet. Differences in gene expression, particularly *RXRA*, ATP binding cassette subfamily G member 1 (*ABCG1*), *NR1H3*, and *Peroxisome Proliferator Activated Receptor Delta (PPARD)*, were evident between MedDiet‐Nuts and the control diet. In the PREDIMED‐Plus trial, no significant differences in gene expression were found between dietary groups. Principal component analysis (PCA) and linear discriminant analysis (LDA) showed overlapping gene expression profiles across different Mediterranean diet interventions. In conclusion, our study highlights the cardiovascular health benefits of long‐term adherence to a Mediterranean diet, both normocaloric and hypocaloric, primarily reflected by mild upregulation of cholesterol efflux‐related genes—specifically involving *RXRA*, *RXRB*, *ABCA1*, *ABCG1*, *Nuclear Receptor Subfamily 1 Group H Member 2(NR1H2)*, and *PPARD*—among elderly adults at high cardiovascular risk. This suggests a potential mechanism by which these diets may exert cardiovascular protective effects.

## Graphical abstract

The graphical abstract (GA) displays the study of two randomized trials studying the molecular mechanisms of cholesterol efflux‐related genes analyzed via transcriptomics after two Mediterranean diet interventions. Cholesterol efflux molecules include ATP‐binding cassette transporters ABCA1 and ABCG1 in cholesterol transport to HDL. It also depicts the transcriptional regulation, key in the cholesterol efflux process, by nuclear receptors (NR1H2, NR1H3, RXRA, RXRB, PPARA, PPARD, and PPARG).

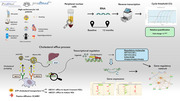

**Abbreviations**
ABCA1: ATP binding cassette subfamily A member 1
ABCG1: ATP binding cassette subfamily G member 1
BP: blood pressure
CAV1: Caveolin‐1
CEC: cholesterol efflux capacity
Ct: cycle threshold
CVD: cardiovascular disease
Er‐MedDiet: energy‐reduced MedDiet
FC: fold‐change
HDL: high‐density lipoprotein
HDL‐c: high‐density lipoprotein cholesterol
LDL‐c: low‐density lipoprotein cholesterol
MedDiet: traditional Mediterranean diet
MedDiet‐EVOO: traditional Mediterranean diet enriched with extra‐virgin olive oil
MedDiet‐Nuts: MedDiet enriched with nuts MedDiet‐Nuts
NR1H2/LXR‐α
: nuclear receptor subfamily 1 group H member 2
NR1H3/LXR‐β: nuclear receptor subfamily 1 group H member 3
PCA: principal component analysis
PPAR: peroxisome proliferator activated receptor
PUFA: polyunsaturated fatty acid
RXRA: retinoid X receptor alpha
RXRB: retinoid X receptor beta
SCARB1: scavenger receptor Class B Type 1
SFA: saturated fatty acid

## 1 Introduction

Cardiovascular diseases (CVDs) are the leading cause of death globally, representing an estimation of 17.9 million of deaths [1]. Lifestyle and dietary patterns are key factors in the development of metabolic syndrome or its components [2, 3]. The traditional Mediterranean diet (MedDiet) is characterized by a high intake of extra‐virgin olive oil, cereals, legumes, fish, vegetables, and fruit [4], along with a lower consumption of red and processed meat. This diet has demonstrated beneficial effects on cardiovascular risk factors by reducing inflammatory biomarker levels and improving the lipid profile, in particular, high‐density lipoprotein (HDL) functionality [5, 6, 7, 8]. HDL function enhancement has been reported under different MedDiet scenarios, such as a 12‐month longitudinal clinical trial with a MedDiet supplemented with EVOO and nuts [8].

In the era of precision medicine, blood transcriptome has been put forward as a surrogate and accessible tissue that allows to infer or predict disease‐related data for different purposes [9, 10], including CVDs and nutrients interaction [11, 12]. Previous PREDIMED substudies have examined blood transcriptome response to dietary interventions supplemented with EVOO or nuts. The effects of a 3‐month MedDiet intervention on the expression of cardiovascular risk‐related genes were reported using whole transcriptome microarray analyses in elderly subjects at high cardiovascular risk (traditional Mediterranean diet enriched with extra‐virgin olive oil [MedDiet‐EVOO] through IL1b, IL1RN, TNF‐α, and ICAM1) [13]. Furthermore, following a 3‐month intervention with the PREDIMED MedDiet enriched with mixed nuts or EVOO, a downregulation of transcriptomic pathways related to neuroinflammation (MedDiet enriched with nuts MedDiet‐Nuts [MedDiet‐Nuts], with downregulation levels of TNF‐α, CCL3, IL‐8, and IL10) was observed [14]. Additionally, after a 3‐month intervention with a traditional MedDiet, particularly when supplemented with virgin olive oil, decreased gene expression linked to inflammation (INF‐γ, ARHGAP15, and IL7R) and oxidative stress (ADRB2, POLK) was observed in healthy subjects [15]. Within the framework of the PREDIMED study, following a long‐term MedDiet intervention (3 years), no statistically significant changes were observed between the MedDiet groups and the control group, whereas the control group showed a tendency to increase the gene expression of two inflammatory receptors involved in the pathogenesis of atherosclerosis (CXCR2, CXCR3) [16].

Based on the association between MedDiet and the overall cardiovascular benefit, along with HDL functionality, we selected a subset of candidate genes involved at different stages of cholesterol efflux, to study the transcriptional landscape. First, ATP binding cassette subfamily A and G member 1 (*ABCA1* and *ABCG1*) are membrane‐bound proteins involved in cholesterol and phospholipid transport that are expressed in multiple tissues, where they play a role in reverse cholesterol transport, HDL lipoprotein formation, and pumping cholesterol to HDL particles at different stages [17]. At the regulation stage, nuclear receptor subfamily 1 group H members 2 and 3 (*NR1H2* and *NR1H3*, also known as *LXRB* and *LXRA*) belong to a superfamily involved in the modulation of reverse cholesterol transport through the translated protein's ability to form partnerships with functionally related molecules to regulate *ABCA1* and *ABCG1* expression [18, 19, 20]. In a similar way, retinoid X receptors (RXRs) can operate as lipid sensors and partner with a variety of molecules to exert a wide range of functions including cholesterol efflux capacity (CEC) promotion [21]. ABCA1, ABCG1, and scavenger receptor Class B Type 1 (*SCARB1*, also called *SR‐BI*) cholesterol transporters are involved in cholesterol efflux from macrophages to lipid‐free apoA‐I and HDL as a first stage of reverse cholesterol transport [22]. Caveolin‐1 (CAV1) is a structural protein of caveolae (or plasma membrane invaginations) involved in cholesterol transport and signaling [23]. Finally, peroxisome proliferator activated receptors (PPARs) are ligand‐transcription factors with upregulating activity in several proteins involved in CEC and reverse cholesterol transport, fat storage, and oxidation [24, 25].

To understand these molecules behavior in well‐established interventions with solid evidence of ameliorating HDL functionality, the aim of the present study was to examine the transcriptomic response of cholesterol efflux‐related genes and assess the long‐term effects after 12 months of different Mediterranean diet interventions in older adult subsamples at high cardiovascular risk.

## 2 Methods

### 2.1 Study Design and Subject Recruitment

Our study population came from two clinical trial samples: PREDIMED (PREvención con DIeta MEDiterránea) and PREDIMED‐Plus. Both studies are randomized, parallel, controlled, and nutritional trials. Baseline characteristics of the subsample volunteers compared to the general population in both studies, PREDIMED and PREDIMED‐Plus, are described in Table S1.

### 2.2 PREDIMED Study

The PREDIMED study was a large‐scale multicenter trial of 7447 participants, that assessed the effect of a supplemented MedDiet on the primary prevention of CVD [26]. The PREDIMED population for our study was a random subsample of volunteers (*n* = 151, 77 women, and 74 men, MedDiet‐EVOO [*n* = 54], MedDiet‐Nuts [*n* = 46], Control [*n* = 51]). The subsample included individuals from seven different recruiting sites, with similar baseline main characteristics (age, sex, hypertension, weight, body mass index [BMI], smoking status, cholesterol, triglycerides, and glucose levels, with the exception of diabetes status proportion). The hypothesis was based on the comparison of two traditional MedDiets, one supplemented with extra‐virgin olive oil (MedDiet‐EVOO), another with nuts (MedDiet‐Nuts), plus a third one as low‐fat diet advice (control group). Participants in the MedDiet group received educational sessions on an ad libitum MedDiet based on a 14‐item nonenergy restricted score [27]. No specific advice for increasing physical activity or losing weight was provided.

Eligible participants of the PREDIMED trial were women aged 60–80 years and men between 55 and 80 years who met at least one of the following criteria: (1) Type 2 diabetes or (2) ≥3 major cardiovascular risk factors, out of the following: current smoking (>1 cig/day during the last month); hypertension (systolic blood pressure (BP) ≥140 mmHg or diastolic BP ≥ 90 mmHg or antihypertensive medication); low‐density lipoprotein cholesterol (LDL‐c) ≥ 160 mg/dL or lipid‐lowering therapy; high‐density lipoprotein cholesterol (HDL‐c) ≤ 40 mg/dL in men or ≤ 50 mg/dL in women; BMI ≥ 25 kg/m^2^; or family history of premature coronary heart disease [28]. Exclusion criteria included: prior history of CVD, severe chronic illness, drug or alcohol addiction, history of allergy or intolerance to olive oil or nuts, a low predicted likelihood of changing dietary habits according to the stages of change model [29], or any condition that could impair study participation [26]. Main subsample characteristics regarding anthropometric measurements, glucose and lipid metabolism, and lifestyle habits including diet and physical activity questionnaire scores are described in Table 1. The data presented in Table 1 include results from both the baseline and 12‐month follow‐up assessments, as well as the changes observed between these two‐time points.

**TABLE 1:** Mean and standard deviation of key lipid and glucose metabolism variables, anthropometric measurements, adherence to diet and physical activity questionnaire scores of PREDIMED at baseline, 12‐month follow‐up, and the change between the two time points.

	MedDiet‐EVOO			MedDiet‐Nuts			Control			Baseline comparison to control		12‐month comparison to control		12‐month change comparison to control	
	Baseline	12 months	12‐ month change	Baseline	12 months	12‐month change	Baseline	12 months	12‐month change	MedDiet‐EVOO	MedDiet‐Nuts	MedDiet‐EVOO	MedDiet‐Nuts	MedDiet‐Nuts	MedDiet‐EVOO
Weight1 (kg)	75.61 (10.31)	75.03 (10.28)	−0.58 (2.47)	81.06 (13.81)	80.74 (13.92)	−0.33 (3.24)	84.23 (12.51)	83.52 (13.88)	−0.71 (4.18)	−**8.55 (**−**13.31–(**−**3.93))**	−5.31 (−8.90–2.57)	−**8.29 (**−**13.50**–(−**3.48))**	−4.77 (−8.80–3.24)	0.26 (−1.28–1.55)	0.54 (−1.22–1.99)
Waist circumference (cm)	99.94 (9.20)	100.27 (8.48)	0.17 (5.56)	101.68 (8.91)	101.79 (10.80)	−0.57 (5.18)	104.96 (9.71)	105.16 (10.78)	0.04 (5.21)	−**5.35 (**−**8.90**–(−**1.14))**	−4.51 (−7.32–0.78)	−**4.71 (**−**8.89**–(−**0.89))**	−5.1 (−8.10–1.37)	0.02 (−2.10–2.34)	−0.8 (−2.91–1.68)
BMI (kg/m^2^)	29.84 (3.65)	29.62 (3.73)	−0.22 (1.02)	29.28 (3.62)	29.18 (3.77)	−0.10 (1.14)	31.37 (3.51)	31.06 (3.87)	−0.30 (1.62)	−**1.57 (**−**2.99**–(−**0.07))**	−**2.53 (**−**3.63**–(−**0.53))**	−1.43 (−2.99–0.11)	−**2.26 (**−**3.53**–(−**0.23))**	0.14 (−0.47–0.65)	0.27 (−0.40–0.80)
Total cholesterol (mg/dL)	209.21 (43.77)	201.13 (40.55)	−4.63 (38.13)	198.86 (39.08)	195.35 (31.15)	−3.10 (32.51)	195.39 (34.49)	191.44 (37.69)	−2.96 (42.58)	11.33 (−2.33–29.98)	2.94 (−12.73–19.68)	8.8 (−7.01–26.38)	1.05 (−11.94–19.76)	0.73 (−19.05–15.71)	−0.7 (−17.46–17.19)
LDL‐c (mg/dL)	129.15 (37.30)	130.10 (38.33)	3.69 (37.64)	120.06 (31.94)	116.03 (27.75)	−2.57 (20.70)	120.38 (31.15)	115.00 (26.38)	−7.18 (27.02)	8.81 (−5.34–22.88)	2.73 (−14.36–13.71)	**17.57 (1.02**–**29.18)**	1.15 (−12.60–14.65)	12.78 (−3.17–24.93)	−0.56 (−7.42–16.65)
HDL‐c (mg/dL)	47.92 (7.45)	45.13 (10.26)	−2.45 (6.82)	48.63 (10.64)	50.97 (12.08)	0.62 (6.57)	48.44 (9.96)	47.74 (11.00)	−0.64 (7.39)	−1.74 (−4.18–3.15)	0.74 (−4.36–4.75)	−1.87 (−7.18–1.95)	1.52 (−2.27–8.73)	−1.47 (−4.86–1.25)	1.49 (−2.06–4.57)
Triglycerides (mg/dL)	136.68 [106.03:172.55]	125.60 [105.46:168.53]	−21.66 (148.82)	125.26 [83.22:178.54]	114.22 [81.43:138.65]	−23.84 (61.27)	112.27 [91.51:159.66]	129.39 [98.07:162.99]	7.96 (52.60)	34.06 (−14.49–81.22)	1.44 (−21.56–40.70)	−1.49 (−18.94–32.40)	−14.55 (−44.44–2.71)	−12.08 (−76.14–16.89)	−**26.07 (**−**58.57**–(−**5.05))**
Triglycerides/HDL‐c	3.65 (4.45)	3.41 (2.07)	−0.28 (4.31)	3.13 (2.25)	2.42 (1.31)	−0.54 (1.64)	2.87 (1.56)	3.10 (1.68)	0.30 (1.36)	0.82 (−0.57–2.14)	0.07 (−0.60–1.13)	−0.01 (−0.49–1.12)	−0.21 (−1.38–0.03)	−0.16 (−1.91–0.75)	−**0.59 (**−**1.58**–(−**0.12))**
Glucose (mg/dL)	125.53 (37.83)	119.79 (39.04)	−7.08 (36.65)	125.84 (41.82)	124.90 (37.26)	−3.85 (36.70)	131.64 (55.12)	117.28 (26.70)	−8.87 (46.24)	−3.76 (−25.86–13.64)	−9.46 (−27.04–15.44)	1.69 (−11.60–16.61)	6.93 (−7.54–22.78)	−2.72 (−16.26–19.84)	7.95 (−14.10–24.12)
Adherence to MedDiet (14‐point item score)	8.57 (2.02)	10.24 (1.41)	1.67 (2.15)	8.62 (2.01)	10.72 (1.57)	2.10 (2.57)	8.98 (1.72)	9.07 (1.68)	0.09 (1.87)	−0.41 (−1.17–0.36)	−0.41 (−1.18–0.45)	**1.16 (0.55**–**1.81)**	**1.63 (0.95**–**2.35)**	**1.57 (0.77**–**2.41)**	**2.04 (1.03**–**3.01)**
Physical activity (MET∙min/week)	1854 (1372)	1623 (959)	−230 (1263)	2232 (1995)	2573 (2233)	341 (1352)	1684 (1650)	2033 (2151)	348 (1601)	208.26 (−450.70–791.56)	441.37 (−252.35–1348.14)	−357.72 (−1099.83–281.78)	487.9 (−410.41–1492.30)	−565.97 (−1170.48–11.57)	46.52 (−644.32–630.42)

*Note*: Median and 1st–3rd quartile range are displayed for triglycerides (non‐normal distributed variable). Mean of differences and confidence intervals from Student's *t* test comparison between groups at baseline, 12‐month follow‐up, and 12‐month change are presented. Bold letters indicate confidence intervals excluding zero.

Abbreviations: BMI, body mass index; HDL‐c, high‐density lipoprotein cholesterol; LDL‐c, low‐density lipoprotein cholesterol; MedDiet, Mediterranean diet; MedDiet‐EVOO, Mediterranean diet supplemented with extra‐virgin olive oil; MedDiet‐Nuts, Mediterranean diet supplemented with nuts.

### 2.3 PREDIMED‐Plus Study

The PREDIMED‐Plus is a multicenter lifestyle intervention with 6874 eligible participants. It is a randomized trial conducted in 23 Spanish centers with a large cohort presenting metabolic syndrome recruited from primary healthcare centers [30]. In the PREDIMED‐Plus trial, the study population was a subgroup of 89 participants randomly selected (39 women and 40 men, intervention group [*n* = 44], Control group [45]) from the IMIM (Hospital del Mar Research Institute) recruiting site. Inclusion criteria were men aged 55–75 years and women 60–75 years, with overweight/obesity (BMI: 27–40 kg/m^2^) and meeting at least three metabolic syndrome components at baseline: (1) triglycerides ≥150 mg/dL or triglyceride‐lowering medication; (2) fasting glucose ≥100 mg/dL or glucose‐lowering medication; (3) systolic/diastolic BP ≥130/85 mmHg or antihypertensive medication; (4) low HDL‐c levels <50 mg/dL in women and <40 mg/dL in men or medication; and/or (5) waist circumference in women and ≥102 cm in men [31, 32].

Participants were randomly assigned to either an energy‐reduced MedDiet (Er‐MedDiet) intervention or an ad libitum MedDiet control group. Those in the active intervention group followed an Er‐MedDiet with physical activity promotion and behavioral support to meet specific weight loss objectives. The participants received recommendations based on a 17‐item MedDiet score [33]. In addition, physical activity counseling to gradually increase exercise intensity to 150 min/week and attitudinal lifestyle advice through frequent sessions with dietitians (both individual and collective) were provided. As used in the PREDIMED study, participants in the control group received educational sessions on an ad libitum MedDiet based on a 14‐item nonenergy restricted score, and no specific advice for increasing physical activity or losing weight was provided. Main subsample characteristics regarding anthropometric measurements, glucose and lipid metabolism, and lifestyle habits including diet and physical activity questionnaire scores are described in Table 2. The data presented in Table 2 include results from both the baseline and 12‐month follow‐up assessments, as well as the changes observed between these two‐time points.

**TABLE 2:** Mean and standard deviation of key lipid and glucose metabolism variables, anthropometric measurements, adherence to diet and physical activity questionnaire scores of PREDIMED‐Plus at baseline, 12‐month follow‐up, and the change between the 2 time points.

	Er‐MedDiet baseline	Er‐MedDiet 12m	Er‐MedDiet 12m‐change	MedDiet baseline	MedDiet 12m	MedDiet 12m‐change	Baseline	12 months	12 month‐change
Weight (kg)	88.38 (14.87)	80.30 (14.54)	−8.09 (3.31)	88.99 (9.83)	86.14 (9.86)	−2.84 (4.14)	−0.6 (−6.14–4.94)	−**5.85 (**−**6.90**–(−**3.65))**	−**5.24 (**−**6.89**–(−**3.60))**
Waist circumference (cm)	109.74 (9.97)	106.13 (9.94)	−3.61 (2.15)	112.46 (8.21)	111.26 (7.94)	−1.20 (2.11)	−2.67 (−6.76–1.32)	−**5.07 (**−**3.47**–(−**1.58))**	−**2.4 (**−**3.36**–(−**1.47))**
BMI (kg/m^2^)	32.28 (3.02)	29.33 (3.31)	−2.95 (1.11)	33.74 (3.43)	32.66 (3.36)	−1.09 (1.58)	−**1.46 (**−**2.88**–(−**0.04))**	−**3.32 (**−**2.56**–(−**1.33))**	−**1.86 (**−**2.47**–(−**1.26))**
Total cholesterol (mg/dL)	232.80 (49.35)	220.41 (42.23)	−12.39 (35.35)	217.12 (35.94)	217.37 (38.80)	0.24 (38.10)	15.68 (−3.29–34.66)	3.05 (−20.39–8.34)	−12.63 (−28.79–3.52)
LDL‐c (mg/dL)	148.63 (45.05)	139.65 (35.68)	−9.74 (32.15)	133.74 (31.78)	133.59 (31.74)	0.49 (32.09)	10.67 (−2.93–32.72)	5.34 (−15.98–9.48)	−10.57 (−25.01–4.56)
HDL‐c (mg/dL)	48.93 (10.01)	51.78 (10.12)	2.85 (8.00)	52.27 (11.92)	52.05 (10.76)	−0.22 (8.28)	−3.34 (−8.18–1.50)	−0.27 (−1.29–5.27)	3.07 (−0.51–6.65)
Triglycerides (mg/dL)	143 [109:213]	131 [99–183]	−26.29 (81.23)	150 [102:180]	144 [99.2–202]	−3.16 (78.94)	13.93 (−25.70–53.55)	−14.71 (−45.44–5.29)	−28.63 (−63.63–6.36)
Triglycerides/HDL‐c	3.90 (2.48)	2.98 (1.73)	−0.92 (2)	3.42 (2.46)	3.24 (1.71)	−0.18 (2.13)	0.48 (−0.60−1.56)	−0.25 (−1.08 − 0.19)	−0.74 (−1.08 − 0.19)
Glucose (mg/dL)	125.30 (38.63)	115.50 (32.44)	−9.80 (25.13)	116.37 (20.88)	110.79 (16.62)	−5.58 (16.22)	8.17 (−5.90−22.24)	3.85 (−8.94−6.52)	−4.32 (−13.63−4.99)
Adherence to MedDiet (17‐point item score)	7.82 (2.85)	11.14 (2.39)	3.32 (3.24)	7.07 (2.59)	9.91 (2.34)	2.84 (2.91)	0.46 (−0.71−1.63)	1.15 (0.00−2.08)	0.68 (−0.68−2.05)
Physical activity (MET∙min/week)	2842 (2745)	3593 (2535)	751 (2287)	2646 (2418)	3041 (2298)	395 (2121)	281.03 (−871.61−1433.66)	640.42 (−389.26−1349.64)	359.39 (−635.35−1354.13)

*Note*: Median and 1st–3rd quartile range are displayed for triglycerides (non‐normal distributed variable). Mean of differences and confidence intervals from Student's *t* test comparison between groups at baseline, 12‐month follow‐up, and 12‐month change are presented. Bold letters indicate confidence intervals excluding zero.

Abbreviations: BMI, body mass index; Er‐MedDiet, energy‐reduced Mediterranean diet; LDL‐c, low‐density lipoprotein cholesterol; HDL‐c, high‐density lipoprotein cholesterol; MedDiet, Mediterranean diet.

### 2.4 Cardiovascular and Lifestyle Factors in PREDIMED and PREDIMED‐Plus Studies

Dyslipidemia was defined as meeting any of the following criteria: HDL‐c < 40 or 50 mg/dL (for men and women, respectively), LDL‐c > 100 mg/dL, triglycerides > 150 mg/dL, or taking any lipid‐lowering drugs [34].

Adherence to diet was assessed with a previously validated 14‐item questionnaire used in the PREDIMED Study [27, 35]. Additionally, a 17‐item energy‐restricted diet questionnaire was adapted from the 14‐item one for the energy‐restricted intervention in PREDIMED‐Plus. PREDIMED‐Plus participants reported their physical activity level through the Regicor Short Physical Activity Questionnaire [36], a validated version adapted from the Minnesota leisure time physical activity questionnaire [37] which was employed for PREDIMED participants.

### 2.5 Blood Chemistry Analysis

Sample collection was performed after an overnight fasting period at baseline and 12‐month follow‐up. Venous blood samples were respectively collected in K3‐EDTA anticoagulant to yield plasma in PREDIMED, and vacuum tubes with a silica clot activator for serum in PREDIMED‐Plus (Becton Dickinson, Plymouth, UK). Serum tubes were centrifuged after the completion of the coagulation process, and plasma tubes immediately after collection, both for 15 min at 1.700 × *g* room temperature. The following analytes were quantified in serum with an ABX Pentra‐400 auto‐analyzer (Horiba‐ABX, Montpellier, France): glucose (mg/dL), triglycerides (mg/dL), HDL‐c (mg/dL), and total cholesterol (mg/dL). LDL‐c was calculated according to the Friedewald formula whenever triglycerides were < 300 mg/dL.

### 2.6 RNA Extraction, Reverse Transcription, and Gene Expression Quantification

Gene expression related to receptors involved in cholesterol efflux including nuclear receptors (retinoid X receptor alpha [*RXRA*], retinoid X receptor beta [*RXRB*], *NR1H2*, *NR1H3*, *PPARA*, *PPARD*, and *PPARG*), membrane transporters (*ABCA1*, *ABCG1*, and *SR‐B1*), and structural receptors (*CAV1*) were established.

Blood samples were collected, at baseline and 1‐year postintervention, and stored at −80°C until further analysis. Nuclear cells were isolated from peripheral blood by using tubes for purification of intracellular RNA from human whole blood (range of white blood cells 4.8 × 10^6^–1.1 × 10^7^ leukocytes/mL) for in vitro diagnostics applications (PAXgene Blood RNA Tube, BRT). RNA concentration (A260) and purity were calculated spectrophotometrically (NanoDrop ND‐1000; NanoDrop Technologies). RNA integrity was assessed by using microcapillary gel electrophoresis (Bioanalyzer, NanoChip; Agilent Technologies) and the RNA integrity number value was calculated with Agilent 2100 Expert Software (Agilent Technologies). Samples were selected with RNA integrity number above 7.

Preamplification step was intended to increase low‐input samples, whose concentration lied between 50 and 200 ng/µL. Recommended target levels were above 200 ng/µL and were obtained using TaqMan PreAmp Master Mix (Applied Biosystems). Reverse transcription to cDNA was carried out with High‐Capacity cDNA Reverse Transcription Kit with RNase Inhibitor (Life Technologies). Microarray RT‐PCR step was performed using QuantStudio 12K Flex Real‐Time PCR System (Life Technologies) and TaqMan OpenArray Real‐Time PCR Master Mix (Applied Biosystems). Subsequently, yielded results were analyzed with QuantStudio 12K Flex Software.

### 2.7 Normalization, Relative Quantification and Gene Expression Change

We employed a relative quantification approach to present the analysis of gene expression data. We tested multiple candidates as possible control genes, allegedly being unaffected by the treatment conditions (known as reference genes). Reference genes used for normalization were selected following the geNorm algorithm running a preliminary analysis to discriminate among 21 candidates, selecting *gapdh* due to the higher stability displayed. To study the differences between baseline value and 12‐month follow‐up, we compared the difference between cycle threshold (Ct) and 12‐month follow‐up minus baseline values (∆Ct). In compliance with the premise that the efficiency of target and reference genes are approximately equal (100% ± 10%) [38, 39, 40], we applied the 2^−ΔΔCt^ method to quantify the change in gene expression. Therefore, the data is presented as a fold‐change (FC) value normalized to the reference gene and relative to baseline value. Each pair of patient samples was allocated in the same plate to remove potential run‐to‐run variation [41, 42, 43, 44, 45, 46, 47].

### 2.8 Statistics

The assessment of the normality distribution of the variables was performed based on normality probability and box plots. To examine temporal changes and group differences, we performed paired Student’s *t* test to assess temporal changes across the intervention and between independent with respect to ΔCt values. Linear mixed‐effect models were fitted to estimate whether the evolution of ΔCt values differs among groups. These models were further adjusted for possible confounding variables such as age, sex, time, and weight, while individuals were included as random effect factor. Finally, the interaction term between time and intervention group was formulated to assess inter‐group variability across the trial. Inter‐individual variability was contemplated through random intercept. Linear mixed‐effect estimation was carried out with the use of restricted maximum likelihood. Analysis was executed using the lme function from nlme R package [48].

Descriptive statistics (mean and standard values) and comparison were calculated at baseline, postintervention, and 12‐month change to display nutritional parameters, energy intake, and key food components. In the PREDIMED population, both MedDiets were compared using independent Student's *t* test with the control diet, while Er‐MedDiet was compared with MedDiet in the PREDIMED‐Plus population. Mean of differences between groups at baseline, postintervention, and 12‐month change (MedDiet‐EVOO—control and MedDiet‐Nuts—control in PREDIMED; and Er‐MedDiet—MedDiet in PREDIMED‐Plus) were also computed and displayed next to confidence intervals from Student's *t* test comparison (Tables S2 and S3).

Principal component analysis (PCA) was conducted to represent the individuals according to their expression along all genes (*ABCA1*, *ABCG1*, *CAV1*, *NR1H2*, *NR1H3*, *PPARA*, *PPARD*, *PPARG*, *RXRA*, *RXRB*, and *SCARB1*). This analysis aimed to visualize outlier individuals or to depict the three PREDIMED groups (Figure S1). PCA was executed with FactoMineR package. Along with PCA, linear discriminant analysis (LDA) was executed to classify individuals in predefined groups, maximizing between‐class variance and minimizing within‐class (Figure S2). Complete cases were selected for all analyses. The level of confidence established for statistical procedures was 0.95. All analyses were executed using R Statistical software.

### 2.9 Statistical Power and Sample Size

The sample size of 31 and 23 participants allowed at least 80% power to detect a statistically significant difference in *ABCA1* gene expression, among the 3 and 2 groups, within the PREDIMED and PREDIMED‐Plus, respectively, of 0.5 units of the relative quantification log_2_FC, assuming a two‐sided type error of 0.05. A common standard deviation of 0.6 is estimated.

### 2.10 Untargeted Functional Analysis

Canonical pathways modulated by dietary interventions were defined using Ingenuity (IPA, QIAGEN Redwood City, www.qiagen.com/ingenuity), a web‐based software application that identifies biological pathways and functions relevant to biomolecules of interest. Functional analysis was executed using log_2_FC (Figure 3).

## 3 Results

Finally, the present study employed samples from 151 and 89 participants of the PREDIMED and PREDIMED‐Plus studies, respectively. Samples from 3 and 2 participants from the PREDIMED and PREDIMED‐Plus trials were respectively excluded due to atypical gene expression values. Additionally, 14 and 5 values, from the PREDIMED and PREDIMED‐Plus trials, respectively, were not included due to lack of amplification of either the target or control gene.

The mean age was 65.8 (±6.29 years) and 65.5 years (±4.7 years) for the PREDIMED and PREDIMED‐Plus participants, respectively. With respect to participants’ lifestyles at baseline, in both studies, the diet and physical activity questionnaire scores did not show statistically significant differences among groups, and they met the minimal physical activity requirements suggested by the American Heart Association (450–750 METxminxweek^−1^).

Energy intake, nutritional parameters, and key food components are summarized in Tables S2 and S3 for the PREDIMED and PREDIMED‐Plus studies, respectively.

Cardiovascular risk factors stratified per group of intervention of PREDIMED and PREDIMED‐Plus are represented in Table S4. The percentage of participants with hypertension and dyslipidemia is slightly higher in the PREDIMED‐Plus study than in PREDIMED.

### 3.1 Gene Expression

**FIGURE 1:**
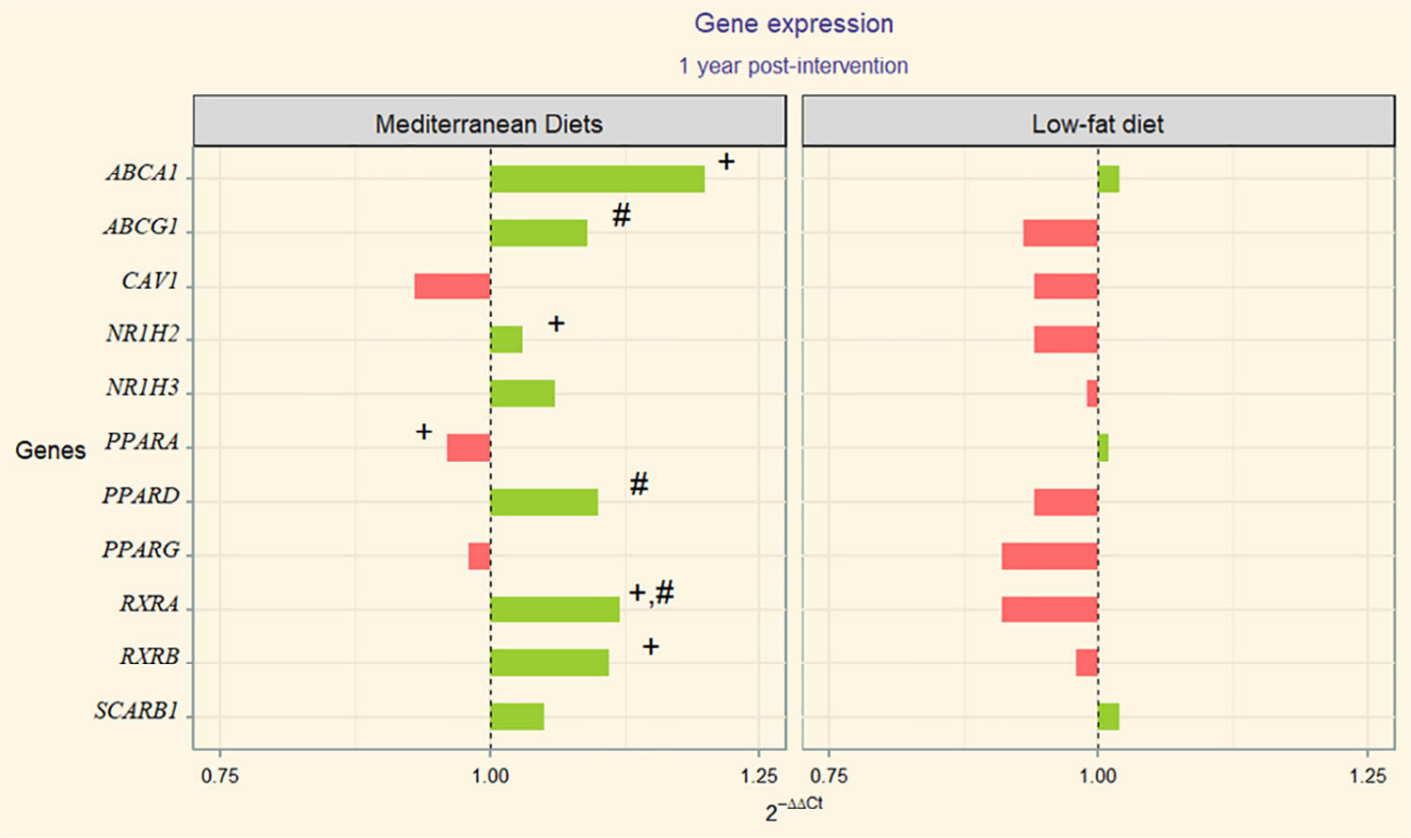
Divergent bar plot depicting the fold‐change mean values per group of PREDIMED (Green color: upregulation and red color: downregulation of genes). Statistically significant (*p* value < 0.05): baseline to postintervention change (paired Student's *t* test) +; time:group interaction (*p* value) from mixed‐effects model compared to control #. Relative quantification (numerical values) of cholesterol efflux‐related genes is presented in Table S5a. Numerical *p* values from statistical analyses are presented in Table S5b.

**FIGURE 2:**
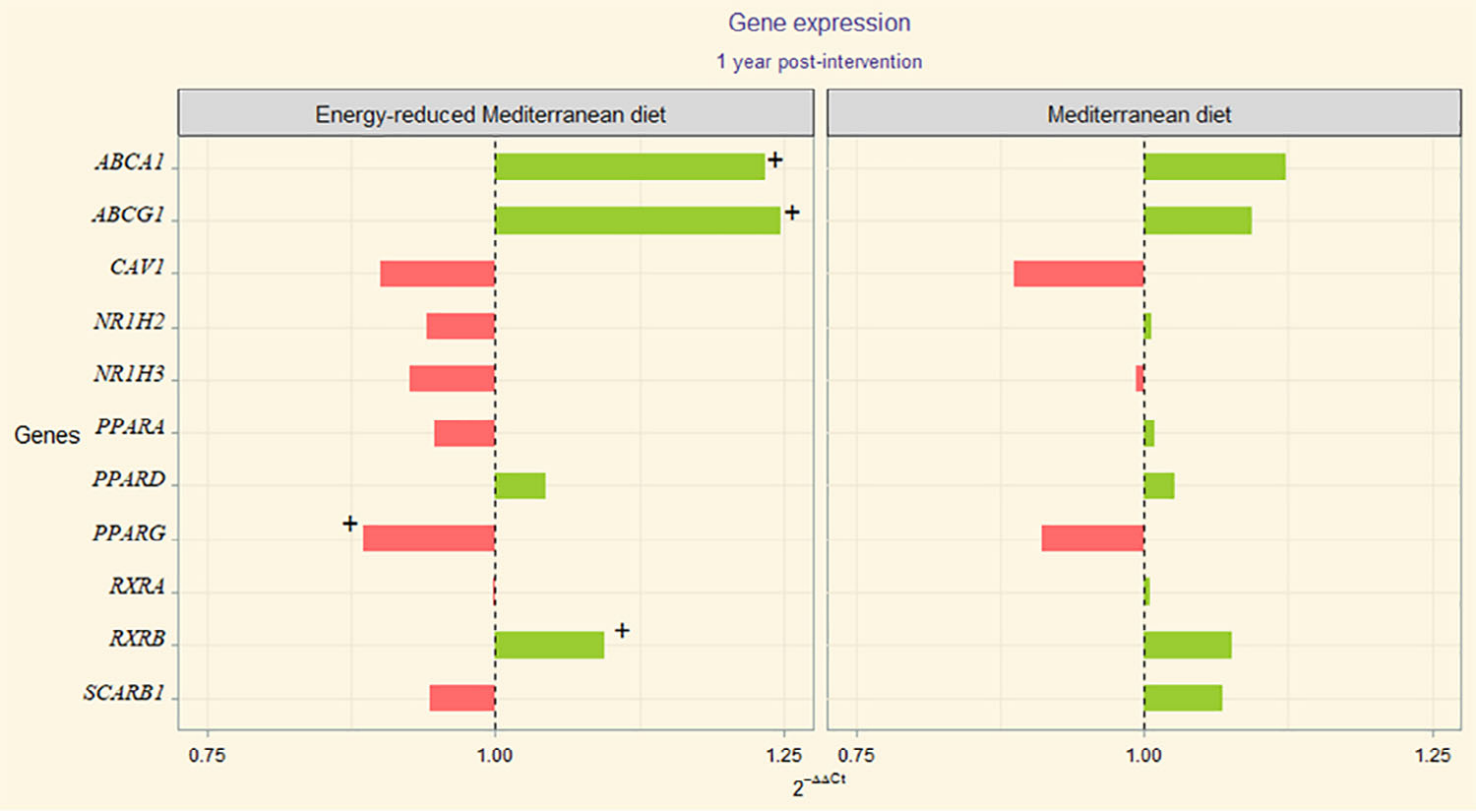
Divergent bar plot depicting the fold‐change (FC) mean values per group of PREDIMED‐Plus (Green color: upregulation and red color: downregulation of genes). Statistically significant (*p* value < 0.05): baseline to postintervention change (paired Student's *t* test) +. Relative quantification (numerical values) of cholesterol efflux‐related genes is presented in Table S6a. Numerical *p* values from statistical analyses are presented in Table S6b.

2^−ΔΔCt^ values correspondent to gene expression patterns (upregulation or downregulation) were depicted through divergent bars chart of both study populations (Figures 1 and 2, PREDIMED and PREDIMED‐Plus respectively and its correspondent numerical values are collected in Tables S5a and S6a).

***PREDIMED Study***: Significant changes occurred along time for baseline to endpoint comparison between baseline and postintervention using Student's *t* test (baseline to 12 months intragroup comparison, Table S5b). These changes occurred in *ABCA1* and *RXRA* in MedDiet‐EVOO and MedDiet‐Nuts, plus *RXRB* in MedDiet‐EVOO, and *NR1H3* in MedDiet‐Nuts group.

On the other hand, when conducting Student's *t* test comparison between independent groups (baseline to 12 months intragroup comparison, Table S5b) yielded statistically significant differences between *RXRA* genes between MedDiet‐EVOO and control groups (*p* value = 0.006). In the case of the MedDiet‐Nuts and control groups, our analysis revealed differences in *RXRA* (*p* value = 0.011) and *PPARD* (*p* value = 0.034). Linear mixed‐effects models adjusted for sex, age, and weight resulted in statistically significant differences for the interaction term time‐group (linear mixed‐effects model time:group term (*p* value), Table S5b) of intervention in *RXRA* (*p* value = 0.009) between MedDiet‐EVOO and control. In the comparison between MedDiet‐Nuts and control, we observed statistically significant results in *RXRA* (*p* value = 0.006), *ABCG1* (*p* value = 0.038), *NR1H3* (*p* value = 0.036), and *PPARD* (*p* value = 0.023). In concordance, in the analysis joining both MedDiet groups, *ABCG1* (*p* value = 0.048), *PPARD* (*p* value = 0.036), and *RXRA* (*p* value = 0.002) were differently expressed versus the control group.

*PREDIMED‐Plus Study*: Comparison between baseline and endpoint results yielded statistically significant changes when performing independent Student's *t* test (baseline to 12 months intragroup comparison, Table S6b). These changes occurred after Er‐MedDiet in *ABCA1* and *ABCG1, PPARG*, and *RXRB*. No statistically significant values resulted from linear model or independent Student's *t* test between arms (intergroup comparison and linear mixed‐effects model time:group term [*p* value], Table S6b). No differences were observed in gene expression when comparing subjects with the most extreme outcomes regarding atherogenic dyslipidemia in both groups in the PREDIMED‐Plus study.

The LDA model demonstrated an overall poor classification performance when utilizing the expression values of the selected genes. When applied to PREDIMED, classified correctly 45.34% of individuals using the selected genes and their relative expression values as predictor variables. Cross‐validated table for the classification of PREDIMED groups using combined MedDiets shown in Table S7. When applied to PREDIMED‐Plus trial the estimated classification accuracy was reduced to 32.2% for MedDiet and Er‐MedDiet groups. Cross‐validated table is shown in Table S8.

PCA and LDA performed in PREDIMED and PREDIMED‐Plus are depicted in Figures S1 and S2. The PCA method displayed an overlapping profile of gene expression among the individuals in the different groups. Probability density function plots were depicted for combined MedDiets versus control in PREDIMED, and MedDiet versus Er‐MedDiet in PREDIMED‐Plus, according to the selected genes. The density probability functions overlapped in a broad area of both curves.

**FIGURE 3:**
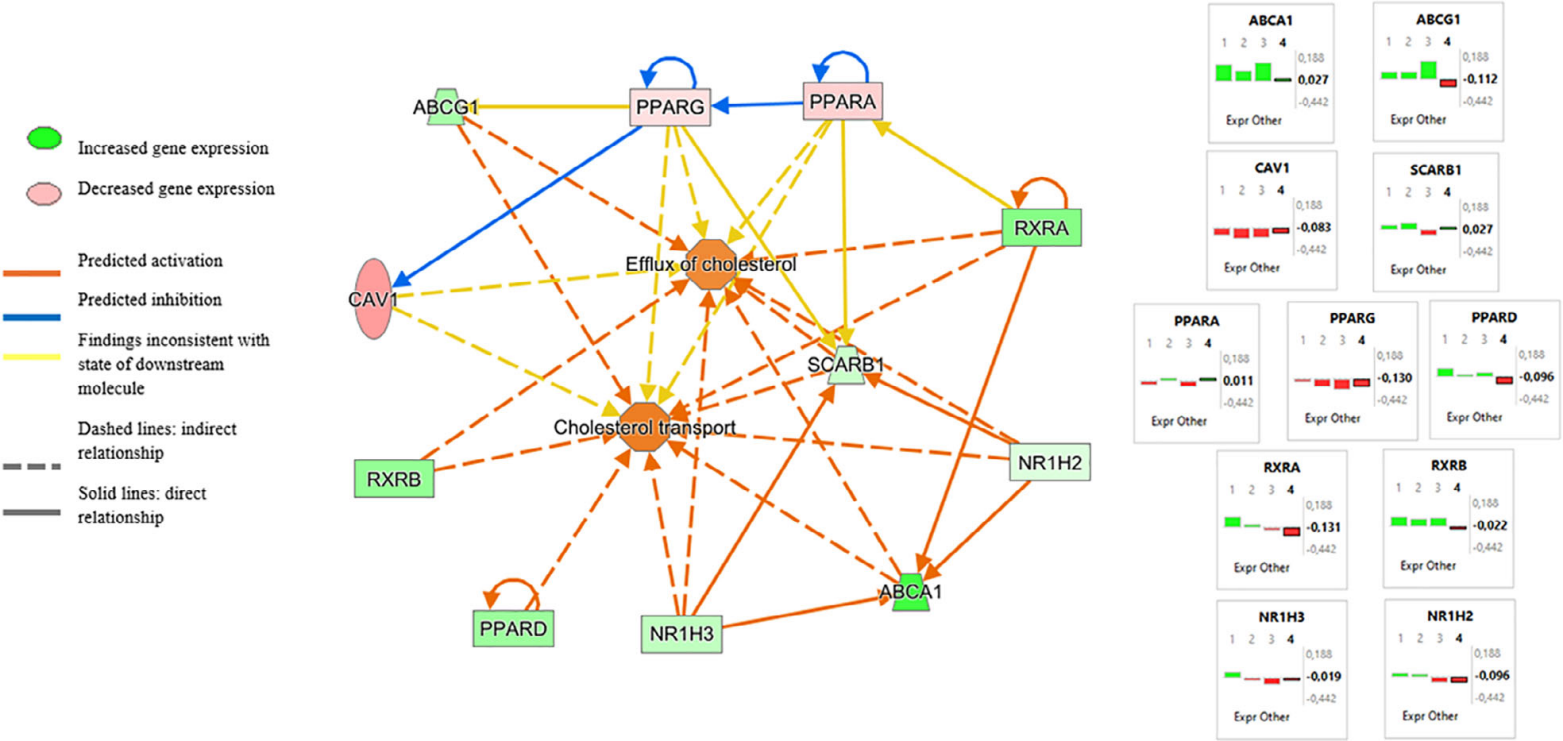
Ingenuity pathway analysis regulatory network prediction using log_2_FC. Legend illustrates the relations of molecules in functional processes related to cholesterol homeostasis. 1: combined MedDiets (PREDIMED), 2: MedDiet (PREDIMED‐Plus), 3: ErMedDiet (PREDIMED‐Plus), 4: Control diet (PREDIMED); Expr Other = ΔΔCt.

Pathway analysis illustrated the relationships between biological functions efflux and transport of cholesterol with the selected gene set: *ABCA1*, *ABCG1*, *NR1H2*, *NR1H3*, *PPARA*, *PPARD*, *RXRA*, *RXRB*, *CAV1*, and *SCARB1*. In accordance with our findings, an upregulation pattern in cholesterol transporters and key regulators was displayed when overlaying the correspondent pathways to the provided nodes. Inconsistent predictions were found for *PPPARG*, *PPARA*, and *CAV1* interaction with cholesterol efflux and transport, as far as the selected genes. A proposed scheme with expression quantification, relationship, and signaling among the different nodes is displayed in Figure 3. The network depiction represents the predicted interactions between the selected molecules using MedDiets combined dataset of PREDIMED. Gene expression values determine the type of interaction (activation, inhibition) among molecules and functions.

## 4 Discussion

We observed an upregulation of the gene expression related to receptors involved in cholesterol efflux function including nuclear receptors (*RXRA*, *RXRB*, *NR1H3*, and *PPARD*), and membrane transporters (*ABCA1, ABCG1*) within the frame of two MedDiet‐intervention trials after 1‐year follow‐up in elderly adults at high cardiovascular risk.

*ABCA1* and *ABCG1* proteins actively participate in the cholesterol removal, the first step of the reverse cholesterol transport. Although *ABCA1* promotes cholesterol movement to nascent HDL particles (lipid‐poor apoA‐I), probably induced by cholesterol‐loaded cells, *ABCG1* and scavenger receptor B1 (*SCARB1*) perform a similar task to larger HDL lipoproteins [17, 49, 50]. Our experiment disclosed baseline to postintervention mild changes in *ABCA1* expression after MedDiet‐EVOO and MedDiet‐Nuts, and also when in the combined MedDiet group. Weight‐adjusted model attested also significant *ABCG1* upregulation when comparing MedDiet‐Nuts and combined MedDiets to the control group. In concordance, within the frame of the PREDIMED‐Plus trial, both groups experienced an upregulation of *ABCA1* and *ABCG1* comparing baseline to endpoint measurements (*p* < 0.01). This study addresses gene expression related to cholesterol transport rather than HDL functionality; therefore, upregulation of key cholesterol efflux genes may not directly reflect into net cholesterol balance.

Lifestyle habits play an influential role in the overall regulation of reverse cholesterol transport, with special interest focused on cholesterol efflux. Dietary pattern's composition has been hypothesized to be a crucial factor affecting CEC gene‐related expression, even though controversial results have been encountered [51], and studies mainly reflect the effects of certain components of the diet, such as fatty acids. The short‐term effects of a high saturated fatty acid (SFA) diet during a 5‐week diet intervention resulted in a downregulation of *ABCA1* and *ABCG1* blood expression, without an increase of plasma inflammatory markers [52]. On the other hand, an upregulation of *ABCG1* blood expression was found after an 8‐week diet intervention replacing a specific quantity of dietary SFA with n‐6 polyunsaturated fatty acids (PUFAs), while maintaining the same monounsaturated fatty acid (MUFA) [53]. In this regard, our findings indicated a common decrease in the consumption of SFA in every group of PREDIMED and PREDIMED‐Plus, along with a higher consumption of MUFA in MedDiet pattern groups (MedDiet‐EVOO and MedDiet‐Nuts in PREDIMED; and MedDiet and Er‐MedDiet in PREDIMED‐Plus), and a slight PUFA increase (in MedDiet‐Nuts), in parallel with an upregulated gene expression of *ABCA1* and *ABCG1* in both studies.

Regarding the impact of physical activity on CEC, the evidence points to an enhanced functionality under moderate or high exercise at midterm [54, 55]. Mid‐term effects have also been evaluated on blood mononuclear cells, yielding an increase in gene expression of *ABCA1* and *ABCG1* during an 8‐week longitudinal study involving low‐intensity exercise [56]. In PREDIMED‐Plus, we observed a larger upregulation in the group engaging in physical activity, Er‐MedDiet, than in the MedDiet traditional group. However, no statistically significant differences were found in comparing groups.

The regulation of *ABCA1* and *ABCG1* constitutes a critical point due to the impact in the overall CEC. This process operates at multiple levels (transcriptional, posttranscriptional, and posttranslational), with a plethora of interrelated molecules participating, forming partnerships, stimulating, and inhibiting each other. Among the most significant we can highlight *NR1H3* also known as *LXRα* (liver‐X‐receptor alpha) and *NR1H2*, also known as *LXRβ* (liver‐X‐receptor beta), homeostasis cholesterol sensors encoding genes, that regulate and actively participate in reverse cholesterol transport [57]. Through obligatory heterodimerization with retinoid X receptors (RXRs), LXRα and LXRβ form a multifunctional partnership susceptible to stimulation by ligands (cholesterol and its metabolites). One of them is the heterodimer LXRα‐RXR working as a major transcriptional regulator of transporters *ABCA1* and *ABCG1*, enhancing cholesterol efflux [20, 50, 58, 59]. With regard to our studies, *NR1H3* showed statistically significant upregulation in MedDiet‐Nuts, versus both the baseline and control group which is a similar pattern of that observed in *ABCG1*. Although we expected a similar pattern among *ABCG1, ABCA1* and *NR1H2, NR1H3*, a negative feedback mechanism after transcriptional stimulation could be present. In addition, different gene expression behaviors might also be attributed to the concentration decrease of cholesterol or its derivative compounds [60]. The lack of correlation between *ABCG1, ABCA1* and *NR1H2, NR1H3* has already been studied in peripheral blood mononuclear cells (PBMCs) samples of hypercholesterolemic patients, under different lipid‐lowering treatments and in controls [61]. In vitro experiments involving human PBMC have led to consider first a differential regulation mechanism between *ABCA1* and *ABCG1*, and secondly, the short span of time of these transcripts upon agonist stimulation [58].

As previously described, RXRs work as transcription factor in various biological processes [24, 58, 62, 63]. Among the natural ligands, different unsaturated fatty acids (docosahexaenoic, linoleic, oleic, and arachidonic acids) and phenolic compounds contained in olive oil have demonstrated activity on RXRs [62, 63, 64, 65]. Previous research has provided insight of how VOO enriched with phenolic compounds, enhanced proteomic expression of LXR/RXR among the top signaling pathways [66]. In concordance, we observed a mild, but statistically significant upregulation of *RXRA* and *RXRB* in the MedDiet‐EVOO participants. Meanwhile, previous studies have pointed out that PUFA contained in nuts mediates the expression liver X receptors [67, 68]. In this regard, our results revealed a significant upregulation of *RXRA* in both MedDiet and combined MedDiet participants, which are also significant in comparison to control.

One of the multiple partners that typically collaborates with RXRs are PPARs, a family of nuclear receptors involved in multiple metabolic pathways related to glucose and lipid regulation, even serving as therapeutic targets (fibrates in PPARα or thiazolidinediones in PPARγ). Differently represented in tissues, the PPARs family is known to intervene in biological processes such as CEC, clearance of oxidized LDL fraction, and reverse cholesterol transport [20, 25, 69, 70]. Previous research with edible oils [73, 74, 75] reported upregulation over time of this PPAR family. In the present study, the overall Mediterranean diet effect in both trials showed mild *PPARD* upregulation, with a significant increase in the MedDiet‐Nuts and combined MedDiet group versus control. Unexpected findings have been reported earlier in referral to *PPARA* expression, after long‐term of single PUFA supplementation [74].

*PPARG* is a well‐known insulin‐sensitizing agent, with already proven activity on lipid metabolism [24, 75]. The PPARγ‐LXRα partnership has been shown to trigger a signaling cascade improving cholesterol efflux [76, 77]. It has been reported physical activity effect would be reflected by an upregulation of *PPARG* [78, 79]. Within our study, the Er‐MedDiet arm, which provided physical activity promotion, there was a significant downregulation in *PPARG* and nonsignificant downregulation in *NR1H3*. This finding might be due to alternative mechanisms regulating *PPARG* [77] or to the fact that the population is elderly with no notable increases of physical activity.

### 4.1 Strengths and Limitations

The first strength of our study lies in its randomized and controlled design, conducted among free‐living individuals. This approach enabled us to generate foundational scientific evidence regarding the effects of the dietary interventions under investigation in the target population. Second, the assembled cohort constitutes a specific group of participants meeting age and risk factors criteria, allowing conclusions to be transferred to analogous population at high‐cardiovascular risk. On the other hand, the same reasoning hinders the possibility to extrapolate to different populations.

Third, peripheral blood cell analysis has been reported as prolific tissue to study CVDs, inflammation, and cholesterol efflux biomarkers. However, it must be taken in account the fact that simultaneous protein analysis has not been performed and could contribute to understanding biological mechanisms. Special attention should be given to the fact that active diet components were not supplemented or provided individually but were incorporated into various sources within a whole diet.

## 5 Conclusion

Mild upregulation of cholesterol efflux‐related genes, involving retinoid X (RXRA, RXRB), ATP‐binding cassette family (ABCG1, ABCA1), liver X (LXRb/NR1H2), and peroxisome proliferator activated (PPARD) receptors, occurred as long‐term responses to different Mediterranean diets in elderly adults at high cardiovascular risk.

## Conflicts of Interest

The authors declare no conflicts of interest.

## Data Availability

PREDIMED: The dataset analyzed during the current study cannot be made publicly available due to national data regulations and ethical considerations, including the absence of explicit written consent from study participants to make their deidentified data available upon study completion. However, data described in the manuscript will be provided to bona fide investigators upon request. Requests can be made by sending a letter to the PREDIMED Steering Committee (predimed‐steering‐committee@googlegroups.com). PREDIMED PLUS: The generation and analysis of the data sets within this study are not projected to be open to access beyond the core research group. This is because the participants' consent forms and ethical approval did not include provisions for public accessibility. However, we follow a controlled data‐sharing collaboration model, as the informed consent documents signed by the participants allowed for regulated collaboration with other researchers for study‐related research. Following an application and approval process by the PREDIMED‐Plus Steering Committee, the data described in the manuscript, alongside the codebook and analytic code, will be available upon request. Researchers interested in this study can reach out to the Committee by sending a request letter to predimed_plus_scommittee@googlegroups.com. For those proposals that gain approval, a data‐sharing agreement, outlining the specifics of the collaboration and data management, will be prepared and finalized. Permission numbers: PREDIMED: ISRCTN35739639; PREDIMED‐Plus: ISRCTN89898870.
